# Identification and characterization of the role of c-terminal Src kinase in dengue virus replication

**DOI:** 10.1038/srep30490

**Published:** 2016-07-26

**Authors:** Rinki Kumar, Tanvi Agrawal, Naseem Ahmed Khan, Yuji Nakayama, Guruprasad R. Medigeshi

**Affiliations:** 1 Vaccine and Infectious Disease Research Center, Translational Health Science and Technology Institute, Haryana, India; 2 Department of Biotechnology, Jamia Hamdard, Hamdard Nagar, New Delhi, India; 3 Department of Biochemistry & Molecular Biology, Kyoto Pharmaceutical University, Kyoto, Japan

## Abstract

We screened a siRNA library targeting human tyrosine kinases in Huh-7 cells and identified c-terminal Src kinase (Csk) as one of the kinases involved in dengue virus replication. Knock-down of Csk expression by siRNAs or inhibition of Csk by an inhibitor reduced dengue virus RNA levels but did not affect viral entry. Csk partially colocalized with viral replication compartments. Dengue infection was drastically reduced in cells lacking the three ubiquitous src family kinases, Src, Fyn and Yes. Csk knock-down in these cells failed to block dengue virus replication suggesting that the effect of Csk is via regulation of Src family kinases. Csk was found to be hyper-phosphorylated during dengue infection and inhibition of protein kinase A led to a block in Csk phosphorylation and dengue virus replication. Overexpression studies suggest an important role for the kinase and SH3 domains in this process. Our results identified a novel role for Csk as a host tyrosine kinase involved in dengue virus replication and provide further insights into the role of host factors in dengue replication.

Dengue virus (DENV) is a mosquito-borne flavivirus, which is estimated to infect 390 million people globally, with 25% of these infections exhibiting disease symptoms each year^1^. Dengue disease manifests wide spectrum of symptoms, from mild dengue fever to severe hemorrhagic form known as dengue hemorrhagic fever (DHF)/dengue shock syndrome (DSS). In addition to antivirals targeting viral proteins directly, identifying host factors required for the virus life cycle provides additional targets for drug development and is an alternate plausible approach to counteract viral infections^2^,^3^,^4^. DENV is a single, positive strand, 11 kb, RNA virus, encoding a single polyprotein that undergoes cleavage by host and viral proteases to form three structural proteins-capsid (C), precursor-membrane/membrane (prM/M) and envelope (E) and seven non-structural proteins (NS1, NS2A, NS2B, NS3, NS4A, NS4B and NS5). The structural proteins constitute the virus particle while the NS proteins are involved in viral RNA replication, virus assembly and modulation of host cell responses^5^.

Tyrosine kinases (TK), comprising of receptor tyrosine kinases (RTK) and cytosolic tyrosine kinases regulate a diverse range of cellular processes from cell division to apoptosis. The human genome encodes 88 TKs and most of the RTKs act as growth factor receptors while cytosolic TKs participate in intracellular signaling by binding to other proteins in response to both extrinsic and intrinsic signals. The structure and function of many of the TKs are well conserved across different species, therefore, many pathogens have evolved to utilize the function of host TKs at various stages of infections thus providing an opportunity to use host TKs as antiviral targets. Drugs targeting host TKs have been in commercial use for conditions such as acute myeloid leukemia, non-small-cell lung cancer, ovarian and other cancers^6^,^7^,^8^. TKs have been shown to be involved at various stages of viral life-cycle. For example, Axl, a receptor tyrosine kinase, was shown to mediate entry of filoviruses^9^. Epidermal growth factor receptor (EGFR) and EphA2 were shown to mediate Hepatitis C virus entry by regulating receptor-co-receptor interactions^10^. siRNA screens and inhibitor studies have identified receptor tyrosine kinases in Influenza virus entry and replication^11^,^12^. In this study we screened a siRNA library targeting human tyrosine kinases to identify TKs that are necessary for infection of DENV in Huh-7 cells. We identified TKs that either inhibited or enhanced DENV infection *in vitro*. We further characterized one of the inhibitors, Csk (c-terminal Src kinase), and show that Csk activity is required for DENV replication and this effect is mediated via regulation of Src family kinases (SFK). Our results identify a new role for Csk in flavivirus replication, which further enhances our understanding of viral replication and provides a new drug target for antiviral development.

## Results

### Identification of tyrosine kinases involved in dengue virus infection

**Figure 1:**
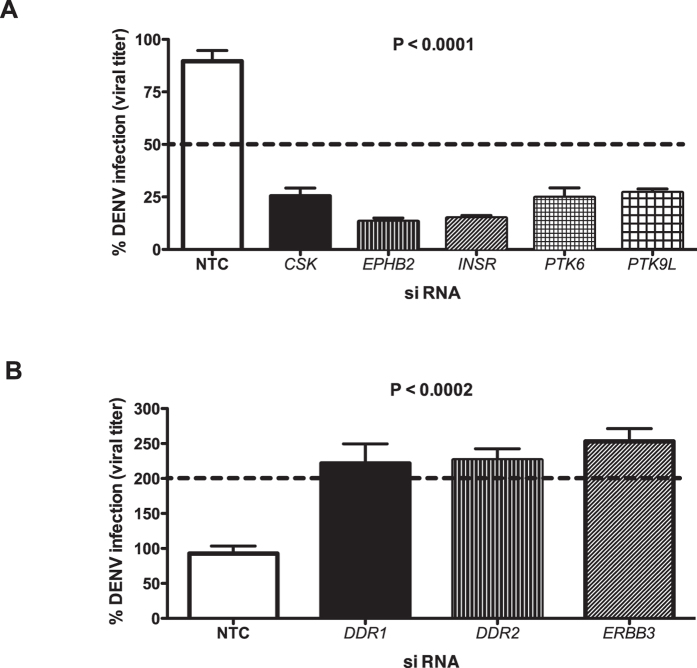
Identification of TKs that inhibit or enhance DENV infection using siRNA screening. Huh-7 cells were transfected with 10 nM of siRNAs targeting the indicated genes and 48 h post-transfection, cells were infected with 1 MOI of DENV2. Viral titers in the infected culture supernatants were measured at 24 h pi by plaque assay. Dotted line indicates 50% titer values relative to non-targeting control (NTC) siRNA in (**A**) and two-fold increase in titers relative to NTC in (**B**). See text for gene abbreviations. The indicated P value was calculated by one way ANOVA using non-parametric Kruskal-Wallis test. The data are from three experiments performed with two or more replicates and indicate mean with SEM (n = 6 to 9).

**Figure 2:**
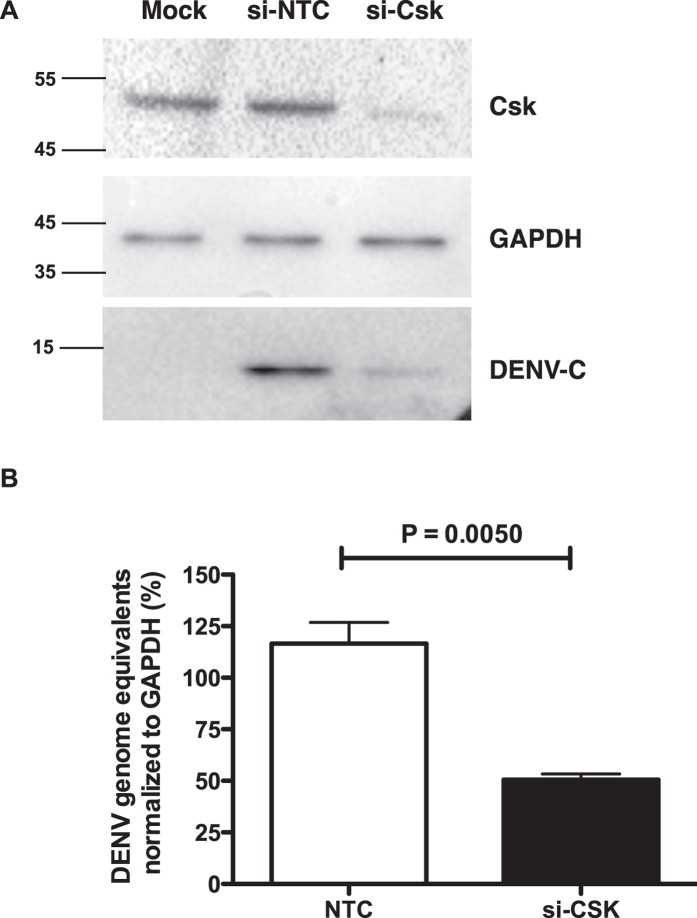
Csk knock-down inhibits DENV replication. (**A**) Huh-7 cells were transfected with 10 nM of siRNAs targeting *CSK* or NTC and 48 h post-transfection, cells were infected with 1 MOI of DENV2. Western blot analysis for Csk, β-actin and DENV capsid protein was performed using cell lysates at 24 h pi. Size of pre-stained molecular weight marker band is indicated. (**B**) RT-PCR analysis to measure the DENV RNA in total RNA isolated at 24 h pi from Huh-7 cells transfected with siRNAs and infected with DENV2 as described above. The data are representative of at least three experiments. Graph represents data from three experiments performed with two replicates each and indicates mean with SEM. P value was calculated by non-parametric, Mann-Whitney test.

**Figure 3:**
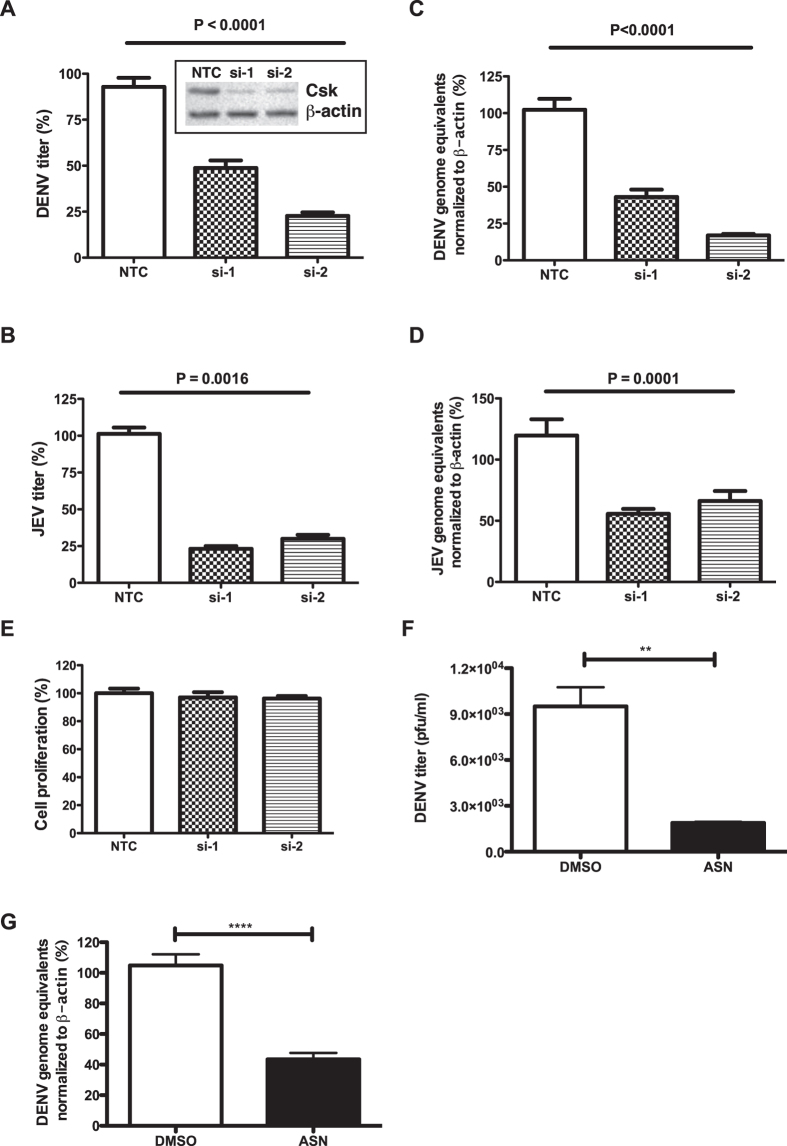
Csk is involved in flavivirus replication. (**A**) Huh-7 cells were transfected with 10 nM of one of the two individual siRNAs targeting *CSK* or NTC and 48 h post-transfection, cells were infected with 1 MOI of DENV2 or (**B**) JEV. Viral titers in the infected culture supernatants were measured at 24 h pi by plaque assay. Inset shows western blot analysis of Csk knock-down efficiency in cell lysates, 48 h post-transfection. β-actin levels is shown as a loading control. (**C**) RT-PCR analysis to measure the DENV RNA or JEV RNA (**D**) in total RNA isolated at 24 h pi from Huh-7 cells transfected with siRNAs and infected with DENV2 or JEV as described above. The data are from three or more experiments each performed with two or more replicates and indicate mean with SEM. P value was calculated by one way ANOVA using non-parametric, Kruskal-Wallis test. (**E**) Huh-7 cells were transfected with siRNAs targeting Csk (si-Csk) or a non-targeting control (NTC) and cell proliferation assay was performed at 48 h post-transfection. UT- Untransfected. Error bars represent mean with SEM. (**F**) Huh-7 cells were pre-treated with DMSO or Csk inhibitor ASN-2324598 for 6 h and infected with DENV-2 at an MOI of 1. Viral titers in the infected culture supernatants were measured at 24 h pi by plaque assay. (**G**) RT-PCR analysis to measure the DENV RNA in total RNA isolated at 24 h pi from cells treated with DMSO or ASN-2324598 and infected with DENV-2 as described above. The data are representative of two experiments performed with three replicates and indicate mean with SEM. P value was calculated by unpaired t test. ****p < 0.0001; **p < 0.005.

To identify human TKs that are involved in dengue virus infection, we used a siRNA library targeting 88 human TKs. Huh-7 cells were transfected with the siRNAs and 48 h post-transfection cells were infected with 1 MOI of DENV-2. Culture supernatants were collected from infected cells at 24 h post-infection (pi) and viral titers were measured by plaque assays. siRNAs that either inhibited the viral titers by 50% or enhanced infection over two-fold relative to a non-targeting control siRNA were considered as hits (see Supplementary Fig. S1). After excluding siRNAs that were affecting cell viability, we identified five TKs namely c-terminal src kinase (CSK), ephrin type B receptor-2 (EPHB2), insulin receptor (INSR), protein tyrosine kinase 6 (PTK6) and protein tyrosine kinase 9-like (PTK9L) that caused a ≥2-fold reduction in DENV titers in the culture supernatants without cytotoxicity. Three TKs, discoidin domain receptor tyrosine kinase 1 (DDR1), discoidin domain receptor tyrosine kinase 2 (DDR2) and v-erb-b2 avian erythroblastic leukemia viral oncogene homolog 3 (ERBB3) caused a ≥2-fold increase in viral titers in the supernatant (Fig. 1A,B). We found that targeting Csk consistently inhibited DENV titers when either smart-pool siRNA (Fig. 2) or at least two different siRNAs were used for knock-down (Fig. 3). Therefore, all further experiments were focused on characterizing the role of Csk in DENV life-cycle.

### CSK is involved at early stages of viral life-cycle

**Figure 4:**
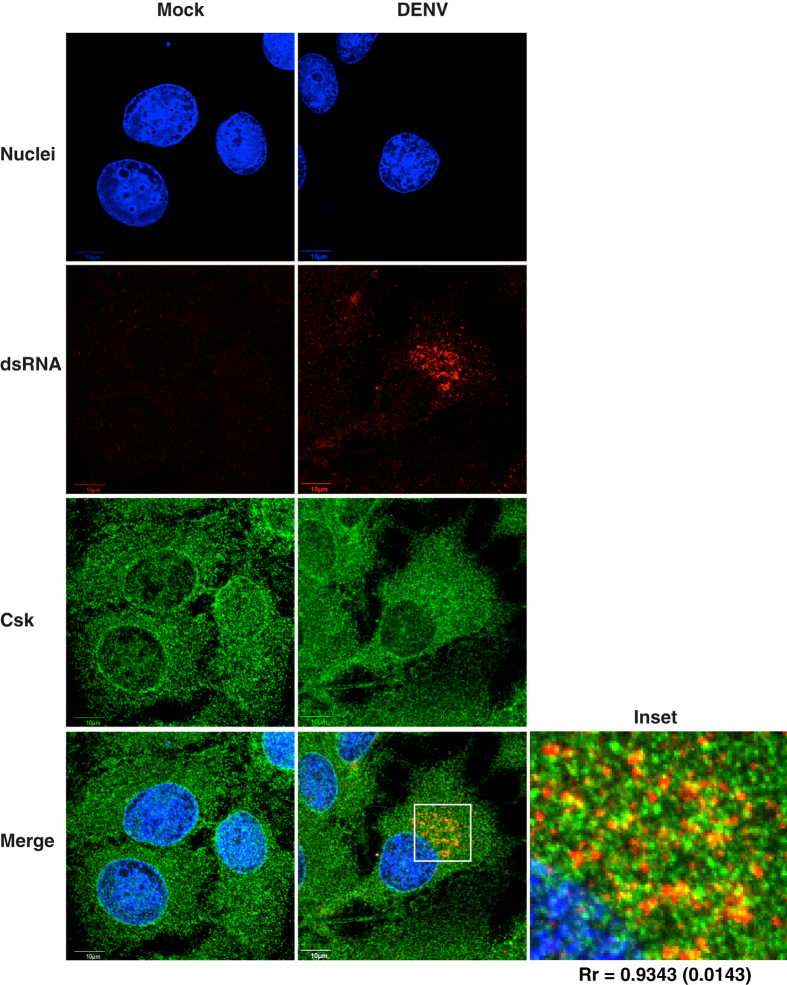
Csk localizes with viral RNA. Huh-7 cells were infected with DENV-2 at an MOI of 5 pfu/cell and at 24 h pi cells were fixed and stained for dsRNA and Csk followed by secondary antibodies conjugated with Alexa-586 (red) and Alexa-488 (green) respectively. Nuclei are stained with DAPI. Scale: 10 μm. Inset shows the blow-up of marked area in merged image. Pearson’s correlation coefficient (Rr) was calculated using Fluoview software. Rr is the average value from ten independent images with SD in parenthesis.

To further identify the stage of viral life-cycle that Csk is involved in, we performed knock-down experiments with Csk siRNA and cell lysates and total RNA was prepared from DENV-infected cells. We found lower levels of DENV capsid expression in cells with reduced Csk expression indicating that Csk inhibits DENV life-cycle at a stage prior to viral protein translation (Fig. 2A). We measured DENV RNA levels by RT-PCR and found that cells transfected with si-CSK had more than 50% reduction in the amounts of DENV RNA as compared to control siRNA (Fig. 2B). To further confirm whether this is a cell-type specific effect or not, we performed knock-down experiments in BHK-21 cells and show that reducing Csk expression in these cells had the same effect on DENV titers as observed in Huh-7 cells (see Supplementary Fig. S2). We next validated these observations by performing experiments with two individual siRNAs from the smartpool against Csk. We found that both siRNAs significantly reduced Csk expression levels and were able to suppress production of infectious virus by inhibiting viral RNA replication (Fig. 3A,C) indicating that the inhibition of viral replication is linked to Csk knock-down and not due to off-target effects of smart-pool siRNAs. To further verify if the observed effect of Csk knock-down is specific to DENV, we infected siRNA-transfected cells with another closely related flavivirus, Japanese encephalitis virus (JEV), and measured viral titers in the supernatants by plaque assay and viral RNA levels in cells by RT-PCR. Csk knock-down showed a similar effect on JEV infection and both viral titers and viral RNA levels were significantly reduced in cells transfected with Csk siRNAs (Fig. 3B,D). We further verified that the observed inhibition was not a result of reduced cell proliferation due to siRNA treatment (Fig. 3E). We next performed virus internalization assays in cells treated with non-targeting control (NTC) or CSK si-RNAs and found no difference in the amount of virus particles entering cells suggesting that the effect of Csk knock-down is at a stage post-viral entry (see Supplementary Fig. S3). Next, we treated cells with a Csk inhibitor, ASN 2324598^13^ followed by infection with DENV. Viral titer and viral RNA levels were measured as described above. We found that inhibiting Csk using a specific inhibitor had similar effects as suppressing Csk expression by siRNAs. Both viral titers and viral RNA levels were significantly reduced in cells treated with Csk inhibitor as compared to mock-treated cells (Fig. 3F,G). These results suggest that Csk plays a role at the stage of viral RNA replication in flavivirus life-cycle. We next performed immunofluorescence analysis to study localization of Csk in DENV-infected cells. Huh-7 cells infected with DENV2 and cells were fixed and stained for Csk and dsRNA at 24 h pi. We found that Csk was uniformly distributed in the cytosol and to discrete membrane compartments and this pattern of localization of Csk was similar in both mock- and DENV-infected cells (Fig. 4). In DENV-infected cells. Some of the viral replication compartments, indicated by presence of dsRNA, were also positive for Csk suggesting that a subset of endogenous Csk localizes to areas of active viral replication (Fig. 4). These observations indicate that Csk plays a role in viral replication either by direct interaction with viral replication machinery or via factors that participate in viral RNA replication.

### Src family kinases are required for regulation of DENV replication by Csk

**Figure 5:**
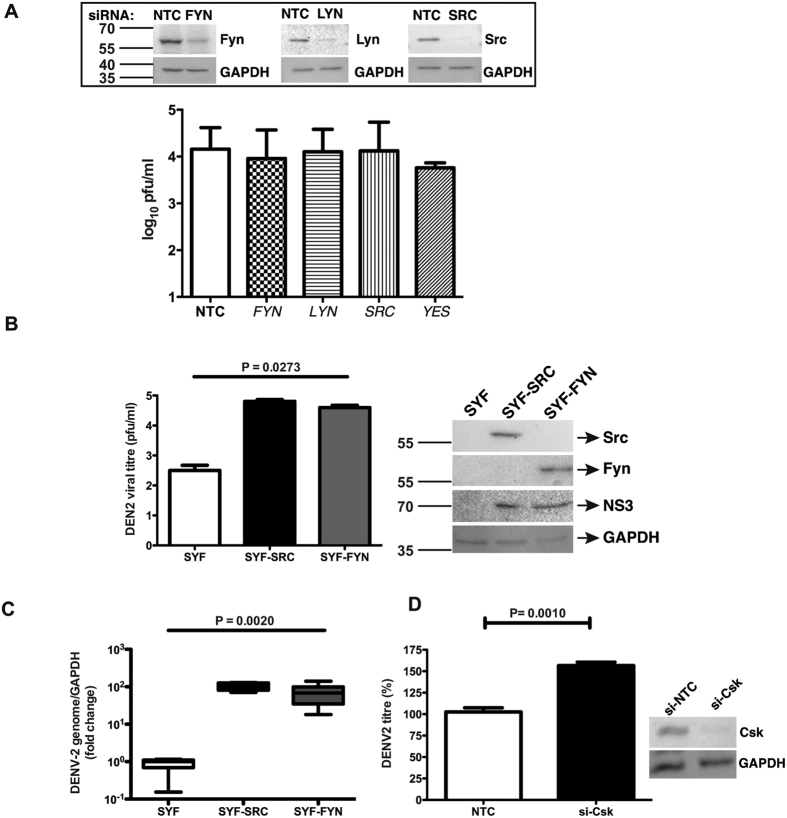
Role of Src family kinases in dengue replication. (**A**) Huh-7 cells were transfected with 10 nM of siRNAs targeting the indicated genes or NTC and 48 h post-transfection, cells were infected with 1 MOI of DENV2. Viral titers in the infected culture supernatants were measured at 24 h pi by plaque assay. Inset shows western blot analysis of knock-down efficiency of Fyn, Lyn and Src in cell lysates, 48 h post-transfection. GAPDH levels are shown as a loading control. Size of pre-stained molecular weight marker band is indicated. (**B**) SYF, SYF-SRC and SYF-FYN cells were infected with 1 MOI of DENV2. Viral titers in the infected culture supernatants were measured at 24 h pi by plaque assay. Western blot analysis of Src, Fyn, NS3 and GAPDH in cell lysates is shown in the side panel. Graph represents one of the three experiments performed in triplicates. P value is calculated by one way ANOVA using non-parametric, Kruskal-Wallis test. Size of pre-stained molecular weight marker band is indicated. (**C**) RT-PCR analysis for dengue RNA levels in infected cells described in B. Graph represents three experiments performed in triplicates. P value is calculated by one way ANOVA using non-parametric, Kruskal-Wallis test. (**D**) SYF cells were transfected with 10 nM siRNAs targeting NTC or *CSK* and 48 h post-transfection, cells were infected with 1 MOI of DENV2. Viral titers in the infected culture supernatants were measured at 24 h pi by plaque assay. P value was calculated by non-parametric, Mann-Whitney test.

Previous studies have indicated that Src family kinases (SFK) mainly, Src and Fyn are involved in DENV infection^14^,^15^. The role of Csk as a negative regulator of SFK signaling is well established^16^. Surprisingly, we did not observe inhibition of DENV infection in Huh-7 cells with any of the SFK siRNAs in our tyrosine kinase library screening (Fig. 5A). This lack of inhibition was not due to insufficient knock-down of SFKs by siRNAs, as we found about 80% reduction in the levels of Fyn, Lyn and Src in siRNA transfected cells by western blotting (Fig. 5A). We speculated that the virus may lack specificity for a particular SFK member and may utilize any available SFK for its function. Therefore, lack of one SFK may not show any effect on virus infection. To confirm this, we utilized mouse embryonic fibroblasts (SYF-MEF) derived from mice lacking the three ubiquitously expressed SFKs (*SRC, FYN, YES*)^17^ and in SYF MEFs in which Src or Fyn expression was restored^18^. MEFs were infected with DENV and viral titers were measured in the supernatant at 24 h pi. We found that DENV titers were about 100 fold less in MEFs lacking the three SFKs as compared to cells expressing either Src or Fyn (Fig. 5B). DENV RNA levels in these cells showed a similar trend suggesting that SFKs are involved at or prior to viral replication stage (Fig. 5C). To next confirm that Csk affects DENV replication via its regulation of SFK activity, we performed Csk knock-down by siRNA in SYF MEFs and infected with DENV to measure viral titers in the supernatant at 24 h pi. Unlike in Huh-7 cells, Csk knock-down had no inhibitory effect on DENV titers in SYF MEFs, on the contrary, suppression of Csk expression in SYF cells significantly enhanced DENV infection (Fig. 5D). This data suggests that Csk expression may also modulate Src-independent pathways or SYF cells may express other kinases that are closely related to SFKs and are regulated by Csk. Overall, our results show that inhibition of DENV replication due to Csk knock-down is linked to SFK signaling.

### Phosphorylation of Csk during dengue virus infection

**Figure 6:**
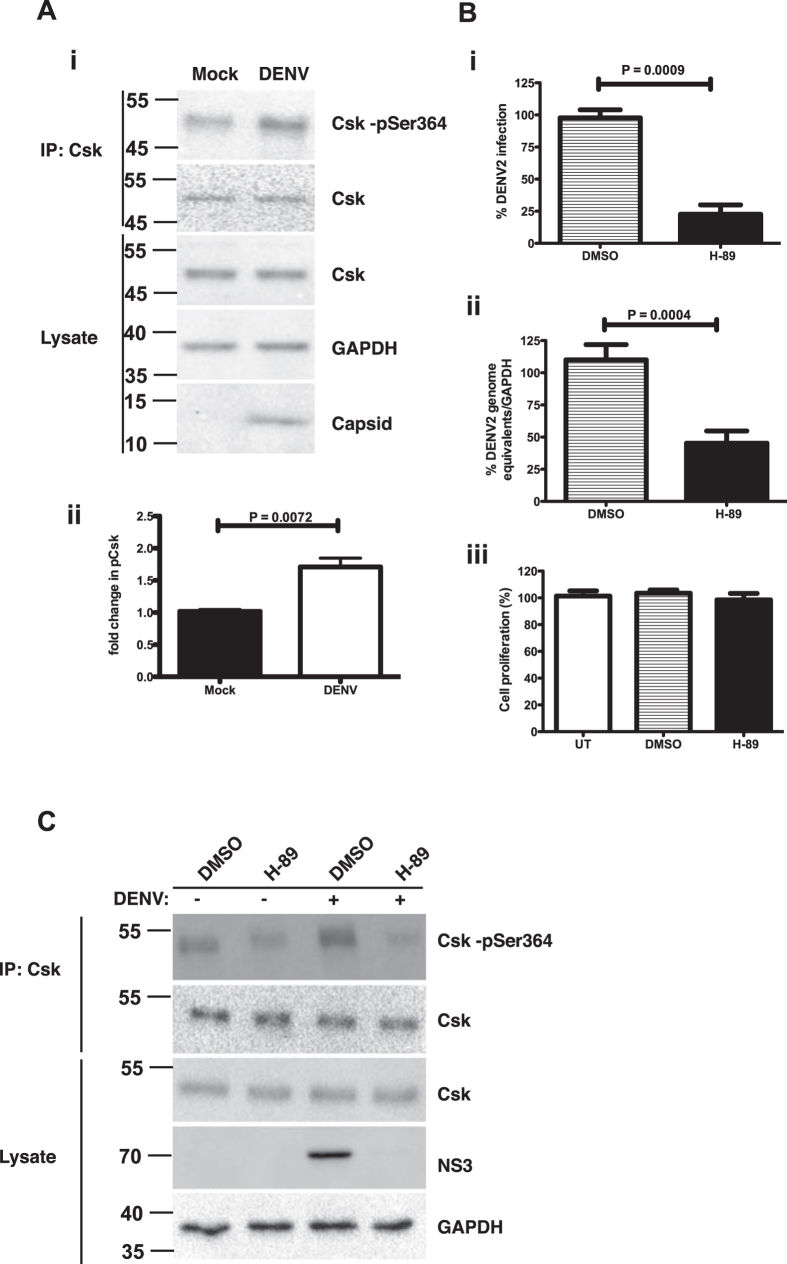
Csk hyperphosphorylation and protein kinase A activity in DENV infection. (**A**) (i) Huh-7 cells were infected with an MOI of 3 pfu/cell of DENV2. Cell lysates were prepared at 24 h pi and Csk was immunoprecipitated. Phosphorylation of Csk was detected by antibody specific to Csk-pSer^364^ by western blot. Total amount of Csk immunoprecipitated was analysed by blotting with Csk antibody. GAPDH in total lysates indicates the equal quantity of protein used for immunoprecipitation. DENV infection was verified by western blotting to detect capsid. (ii) Quantitation of Csk-pSer^364^ western blots is shown. P value was calculated by non-parametric, Mann-Whitney test. Size of pre-stained molecular weight marker band is indicated. (**B**) (i) Huh-7 cells were infected with DENV2 and cells were incubated in medium containing 5 μM of PKA inhibitor, H-89, or DMSO (vehicle control). Viral titers in the supernatant was measured by plaque assays at 24 h pi. (ii) RT-PCR analysis to measure the DENV RNA in total RNA isolated at 24 h pi from Huh-7 cells infected with DENV2 and treated with DMSO or H-89 post-infection. The data are representative of at least three experiments performed with two or more replicates and indicate mean with SEM. P value was calculated by non-parametric, Mann-Whitney test. (iii) Huh-7 cells were incubated in medium containing 5 μM of PKA inhibitor, H-89, or DMSO (vehicle control) and cell proliferation assay was performed at 24 h post-transfection. UT- Untreated. Error bars represent mean with SEM. (**C**) Huh-7 cells were mock-infected or infected with DENV2 and treated with DMSO or 5 μM of H-89 from 1 h pi. Csk was immunoprecipitated at 24 h pi and phospho-Csk was detected by western blot analysis with antibody specific to CSK-pSer364. The amount of total Csk pulled down in immunoprecipitation is shown. Total Csk and DENV-NS3 in the cell lysates is shown. GAPDH levels are shown as loading control. Size of pre-stained molecular weight marker band is indicated.

CSK has been shown to be phosphorylated on Ser-364 by protein kinase A (PKA)^19^. Therefore, we next determined the phosphorylation status of Csk in DENV infection. Csk was immunoprecipitated from infected cell lysates at 24 h pi and the extent of Csk phosphorylation was analysed by immunoblotting with phospho-Csk antibody which recognizes the phosphorylation of Ser -364 residue in Csk. We found a 70% increase in phospho-Csk levels at 24 h pi indicating that DENV infection enhanced phosphorylation of Csk (Fig. 6Ai,ii). We next assessed the effect of inhibiting PKA on DENV infection. Cells were infected with DENV and treated with 5 μM of H-89, a PKA inhibitor, from 1 h pi. Viral titers and viral RNA levels were estimated as described earlier. Inhibition of PKA led to significant reduction in viral titers in the supernatant (Fig. 6Bi) and this effect was due to reduced viral replication as assessed by reduction in the viral RNA levels (Fig. 6Bii). Inhibition of PKA by H-89 did not affect cell proliferation (Fig. 6Biii). To further demonstrate that the effect of PKA inhibition on DENV replication is due to its effect on Csk phosphorylation, cells infected with DENV were treated with DMSO or H-89 at 1 h pi. Csk phosphorylation (pSer-364) was assessed at 24 h pi by immunoprecipitation of Csk from cell lysates. PKA inhibition by H-89 led to reduced phosphorylation of Csk in both mock- and DENV-infected cells. As expected, DENV infection was blocked in cells treated with H-89 as evidenced by reduced expression of dengue non-structural protein 5 (Fig. 6C). These results suggest that phosphorylation of Csk by PKA is potentiated in DENV infection and PKA activity may play an essential role in DENV replication.

### Csk kinase domain and SH3 domain play a role in DENV infection

**Figure 7:**
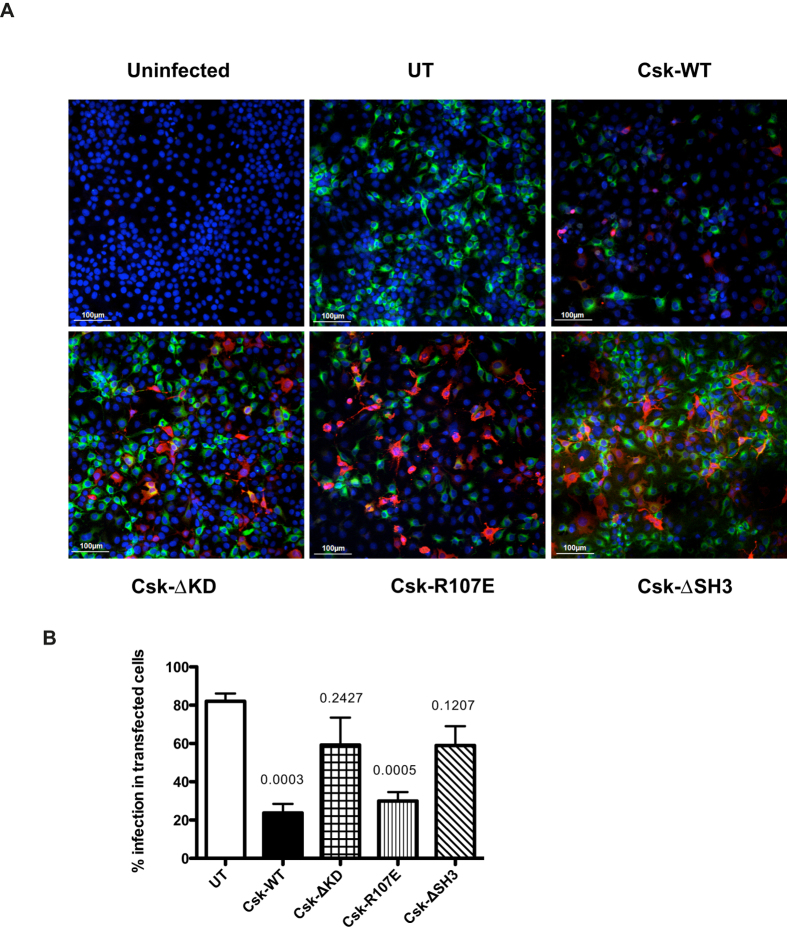
The kinase domain and SH3 domain of Csk is important for DENV infection. (**A**) Huh-7 cells were transfected with the plasmids for overexpression of the indicated proteins tagged with a FLAG epitope for detection. 24 h post-transfection, cells were infected with 3 MOI of DENV2. Cells were fixed at 24 h post-infection and stained using primary antibodies against DENV-Envelope and FLAG epitope followed by secondary antibodies conjugated with Alexa-488 (green) and Alexa-568 (Red) respectively. Nuclei were stained with DAPI. Overlay images are shown. UT- Untransfected. Scale – 100 μm. (**B**) Number of infected cells in untransfected population and in cells expressing the indicated recombinant proteins were counted from each sample. Percentage of infected cells among transfected population relative to UT is shown in the graph. Data represents Mean with SEM values from four independent experiments. P values are indicated which was calculated by unpaired t test between UT and individual transfection condition.

Csk activity is known to be regulated by intra-molecular interactions between different domains of Csk^20^. The SH3 domain of Csk was shown to potentiate the kinase activity of Csk by binding to the phosphorylated kinase domain^21^. We speculated that overexpression of Csk would negatively affect dengue infection by blocking SFK signaling which has been shown to be important in DENV replication^14^. Therefore, expression of wild type (WT) Csk or Csk proteins with mutations or deletions would further help us delineate the region/domain of Csk involved in DENV replication. To test this, we transfected cells with plasmids expressing FLAG-tagged Csk proteins namely; wild-type (Csk-WT), Csk with a kinase domain deletion (Csk-ΔKD), Csk with an inactive SH2 domain (Csk-R107E) and Csk lacking the SH3 domain (Csk-ΔSH3)^22^. Transfected cells were infected with DENV and the efficiency of infection in each of these transfection conditions was analyzed by immunofluorescence. We found that the expression levels of wild type Csk was less compared to other constructs, probably due to pleiotropic effect of dysregulation of SFK signaling pathways as a result of Csk upregulation (see Supplementary Fig. S6). Nevertheless, both the wild type Csk and Csk with a non-functional SH2 (Csk-R107E) domain inhibited virus infection suggesting that overexpression of these proteins has a negative effect on virus infection most likely due to inhibition of SFKs. Both the kinase domain deletion and SH3 domain deletion constructs failed to show the inhibitory effect (Fig. S5; Fig. 7A). We quantitated the number of virus positive cells in untreated cells and in FLAG-positive cells and found a 70–80% reduction in virus-positive cells when Csk-WT and Csk-R107E was expressed. The number of DENV positive cells was not significantly different in cells expressing Csk with a deletion in kinase domain or SH3 domain compared to untransfected cells suggesting that the kinase activity and SH3 domain of Csk are important in DENV infection (Fig. 7B).

## Discussion

The role of TKs in viral infections has been extensively studied. Many viruses require the activity of cellular TKs at various stages of infection^23^. While viruses such as influenza A virus and Kaposi’s sarcoma-associated herpesvirus utilize receptor TKs for entering the cells^11^,^12^,^24^, other viruses like Coxsackievirus depend on the activity of cytosolic TKs for stages post-viral attachment^25^. Ebola virus has been shown to depend on the activity of tyrosine kinases at both entry and later stages of budding and release of virions^26^,^27^. The essential role of TK signaling in viral replication has been identified by a number of studies using multiple approaches such as pharmacological inhibitors of specific TKs, by expression of viruses harboring mutations in TK interaction regions or using siRNA screens against host TKs^12^,^28^,^29^,^30^,^31^,^32^. In most cases, viruses have been shown to activate the cytosolic TK signaling pathways to modulate the cellular environment in favor of virus replication. Viruses influence TK activity either by direct interaction of viral proteins with TKs or by indirectly regulating kinase activity through other mediators. The modulation of TK signaling pathways that activate antiviral innate immune responses in flavivirus infection has been reported earlier^33^,^34^,^35^,^36^,^37^. In addition, Axl, a receptor TK, was shown to mediate the cellular entry of DENV^38^. In the current study, we have identified a new role for Csk in DENV RNA replication. Cells with knock-down of Csk expression by siRNAs showed reduced levels of viral RNA and Csk was shown to partially co-localize with dsRNA suggesting that Csk may either directly associate with the viral replication compartment or may be part of a multi-protein complex that participates in viral replication. We found that Csk was hyper-phosphorylated in DENV infection and inhibiting PKA using a specific inhibitor blocked virus replication suggesting a crucial role for Csk phosphorylation by PKA in DENV replication. Csk activity is regulated by intra-molecular interactions between the SH3 domain and the catalytic domain^20^. In addition, SH3 domain has also been shown to be involved in dimerization of Csk protein which prevents binding of tyrosine phosphatase PEP to the Csk-SH3 domain thus preventing inactivation of SFKs^39^. Overexpression of either the wild type Csk or the SH2 domain mutant of Csk (Csk-R107E) negatively affected DENV infection whereas Csk with a deletion in the kinase domain or the SH3 domain was no longer capable of inhibiting DENV infection. This clearly indicates that the Csk activity and the SH3 domains play an important role in DENV replication.

Src family kinases Fyn and Src were identified as necessary players in DENV RNA replication and assembly respectively^14^,^15^. Another ubiquitous SFK, Yes, was shown to be involved at later stages of WNV life-cycle^40^. However, the exact mechanism of the action of SFKs in dengue virus infection has not been clearly demonstrated. Notably, these studies have shown the role of SFKs in dengue virus replication or assembly/egress by using inhibitors of SFKs which have been shown to target all SFKs^41^,^42^. In our study, SYF cells lacking all three ubiquitously expressed SFKs namely Src, Fyn and Yes showed a drastic reduction in DENV production suggesting that these kinases are essential for DENV replication. We further demonstrated that expression of either Src or Fyn in SYF cells was sufficient to compensate for the absence of the three SFKs suggesting lack of specificity for SFKs in DENV replication. This also explains for the lack of effect on DENV infection when expression of any one of the SFKs were suppressed by siRNAs in Huh-7 cells in our study. Interestingly, a previous study using siRNA screen to identify protein kinases in hepatitis C virus replicon propagation identified Csk as one of the hits and showed that Csk mediated its effect on replication of HCV replicons by suppressing Fyn activity^32^. Whether Csk activity is increased in DENV infection and if there is a dynamic regulation of SFK activity at different stages of DENV life-cycle needs to be examined.

It has been suggested that Csk may be more critical for attenuating SFK activation rather than controlling basal activity of SFK^43^. It is possible that the depletion of Csk probably affects the attenuation of SFK signaling which in turn has an adverse effect on DENV replication. Similarly, overexpression of Csk may also block SFK signaling which negatively impacts viral replication as SFK signaling has been shown to be important for dengue replication^14^,^15^. Negative regulation of SFKs has been the most well-characterized function of Csk, however, recent reports have deciphered a consensus sequence for Csk phosphorylation and suggest that Csk may phosphorylate a number of other cellular proteins^44^. The immunoreceptor tyrosine-based inhibitory motif (ITIM) of platelet endothelial cell adhesion molecule 1 (PECAM-1) and sialic acid binding lectin-like molecule-9 (Siglec-9) was also shown to be phosphorylated by Csk, although this phosphorylation required a prior phosphorylation by SFKs^45^. Sequential phosphorylation of ITIMs was proposed to down-regulate the activity of immunoreceptor tyrosine-based activation motif (ITAM)-dependent signals by recruiting tandem SH2-domain containing tyrosine phosphatases. In addition, as CSK is also known to regulate SFK activity by recruiting tyrosine phosphatases, it would be interesting to investigate the role of tyrosine phosphatases that interact with Csk in DENV replication. Our study has identified a role for Csk and PKA signaling in mosquito-borne flavivirus replication and provides a new perspective into the role of SFK signaling in flavivirus infection. Further efforts to characterize the exact role of Csk in DENV replication may provide novel targets to develop antivirals for dengue disease.

## Materials and Methods

### Cells and Viruses

Huh-7 cells were grown in Dulbecco’s modified Eagle medium (DMEM) supplemented with 10% fetal bovine serum (FBS), 2 mM L-glutamine, 100 units/ml penicillin G sodium and 100 μg/ml streptomycin sulfate and non-essential amino acids at 37 °C and 5% CO_2_. Porcine kidney (PS) cells and BHK-21 cells were grown in the minimal essential medium (MEM) containing 10% FBS, Earl’s salts and additives as mentioned above. SYF and SYF-Src cells were from ATCC. SYF-Fyn cells was described earlier^18^. JEV and DENV strains used in this study have been described previously^46^.

### siRNA transfection

siRNA library targeting the human tyrosine kinase genes were purchased from Dharmacon (Cat. No. G-103100). All transfections were performed by reverse transfection procedure as per manufacturer’s instructions using Lipofectamine RNAimax reagent (Life Technologies). 10 nM of siRNAs were mixed with OptiMEM (Life technologies) and 1 μl of Lipofectamine RNAimax to a total volume of 100 μl in a 24-well plate. Cells were trypsinized and volume made up so as to contain 40,000 cells in 400 μl antibiotic-free medium. After 20 min incubation of the transfection complex, 400 μl cell suspension was added into each well. Each siRNA transfection was performed in triplicates. A group of 10 siRNAs were used for each set of experiments. Knock-down levels were monitored by RT-PCR at 48 h post-transfection from a representative siRNA for each set of experiments and we consistently observed a reduction of 80% or more in the mRNA levels of the target genes under these conditions.

### Plasmids and Antibodies

Csk plasmid constructs used in this study were a kind gift from Lars Rönnstrand^22^. Csk (rabbit) antibody was purchased from Santa Cruz Biotechnology (sc-286) and mouse monoclonal Csk antibody was from BD Transduction Labs (Cat. No. 610079). Rabbit phospho-Csk (p-serine 364) antibody was from Sigma-Aldrich (Cat. No. SAB4503861). Src antibody was from Epitomics (Cat. No. 3795-1). Anti-dsRNA antibody (J2) was from Scicons. β-actin (catalogue number A2228) and anti-FLAG antibodies was purchased from Sigma-Aldrich (Cat. No. F7425). Tubulin antibody was purchased from Developmental Studies Hybridoma Bank (http://dshb.biology.uiowa.edu), DENV Capsid and NS3 antibody has been described before^46^,^47^, GAPDH antibody was purchased from Abcam (Cat. No. AB37168). Horseradish peroxide (HRP)-conjugated IgG raised in goat was used as secondary antibody (Santa Cruz or Life technologies) in western blotting.

### Viral titers, real time PCR and western blotting

Virus growth curve experiments were performed in Huh-7 cells and viral titers in the culture supernatants were determined by plaque assays on BHK-21 cells (for DENV) or porcine kidney cells (for JEV) as described previously^46^. Viral entry assays were performed by infecting cells with 5 pfu/cell of DENV-2. Cells were collected by trypsinization at 1 h pi in complete medium. For quantitation of RNA, cells were washed once with PBS and collected in 250 μl of Trizol reagent (Life Technologies) for isolation of total RNA. Real time PCRs were performed by using one-step or two-step methods using SYBR green or Taqman chemistry as described previously^46^. For immunoblotting, cell lysates were prepared in lysis buffer (50 mM Tris-Cl pH-8.0, 150 mM NaCl, 1% Triton, 0.5% sodium deoxycholate, 0.1% SDS) containing phenylmethylsulfonyl fluoride (PMSF) and protease inhibitor cocktail (PIC) (Roche). Supernatants obtained after centrifugation at 17,000× g were used for loading onto SDS-PAGE and analysed by western blotting as described previously^48^.

### Inhibitor experiments

Huh-7 cells were treated with 20 μM Csk inhibitor (ASN-2324598) for 6 hours and infected with DENV2 at a multiplicity of infection (MOI) of 1. After virus adsorption (1 h), cells were grown in medium containing 20 μM inhibitor or equal volume of DMSO for 24 h. For H-89 treatment, medium containing 5 μM H-89 or equal volume of DMSO was added only after virus adsorption for 1 h. Viral titers in the supernatants collected at 24 h pi were estimated by plaque assay. Cells were trypsinized and collected in 250 μl of defined soyabean trypsin inhibitor (Life technologies) and centrifuged at 200× *g* for 5 minutes. The cell pellet was resuspended in 250 μl of Trizol and processed for real time PCR as described above.

### Immunoprecipitation

Huh-7 cells were infected with DENV2 at an MOI of 5 pfu/cell. Infected cell lysates were prepared at 24 h and 48 h pi in phosphobuffer (Calbiochem) containing PIC and PMSF. Lysates were pre-cleared for non-specific antibody interaction by incubating with rabbit IgG and 30 μl of pre-washed protein A beads at 4 °C for 1 h. Pre-cleared lysates were further incubated with polyclonal Csk antibody overnight at 4 °C. Antigen-antibody complexes were pulled down using protein-A beads and detected by immunoblotting using phospho-Csk-S364 antibody (Sigma-Aldrich). Total Csk immunoprecipitation was quantitated by western blotting with mouse monoclonal Csk antibody.

### Plasmid transfection

FLAG-tagged constructs of Csk were transfected into cells using Lipofectamine 2000 following the manufacturer’s protocol (Life Technologies). Briefly, plasmid DNA and L-2000 were mixed with optiMEM separately and incubated at room temperature for 5 min, and the two were mixed and incubated for 20 min at room temperature. This mixture of DNA and lipid was added to cells plated in antibiotic-free media. After 4 h, media was replaced with complete media. 24 h post-transfection, cells were either infected with DENV2 or further processed for immunofluorescence.

### Immunofluorescence

Cells grown on coverslips were infected with DENV-2 at 5 MOI. 24 h pi, cells were fixed in chilled methanol and processed for immunofluorescence as described previously^48^. Images were acquired using Olympus FLUOVIEW FV1000 confocal microscope with a 20 X or 100 X objective. Images were processed for background subtraction using Fluoview software and prepared for illustration using Image J software (NIH). For colocalization analysis, single slices from Z-stacks were used. A region of interest showing colocalization was marked and extracted. Pearson correlation coefficient of the extracted colocalized pixels was determined using Fluoview software (Olympus). For quantitation of transfection and infection efficiency in plasmid transfections, DENV-E positive and FLAG-positive cells in each image was manually counted in multiple images acquired from each sample.

### Cell proliferation and cytotoxicity assay

Cell proliferation was measured by using Promega’s CellTiter 96^®^ AQ_ueous_ One Solution cell proliferation assay kit as per the manufacturer’s protocol. Briefly, 10,000 cells grown in a 96 well plate were treated with 5 μM H89 or DMSO. After 24 h, 20 μl of the AQ_ueous_ One Solution was added to each well containing 100 μl of media and incubated for 3 h. The reaction was stopped by adding 25 μl of 10% SDS. The supernatant was diluted 1:5 in PBS and absorbance was measured at 490 nm. OD_490_ from media alone with the AQ_ueous_ One Solution was used to subtract background. For cell proliferation assays with siRNA transfected cells, cells were transfected with 10 nM of indicated siRNAs or non-targeting control (NTC) siRNA and cell proliferation was measured as described above at 48 h post-transfection. Cytotoxicity assays were performed as described previously with cells treated as above^48^. Cell viability was determined by trypan blue staining.

### Statistical analysis

GraphPad Prism software was used for all graphical representations and statistical analysis. All experiments were performed in two or three replicate samples and each figure is representative of experiments performed at least three times. Non parametric, statistical tests were performed to calculate P values.

## Additional Information

**How to cite this article**: Kumar, R. *et al*. Identification and characterization of the role of c-terminal Src kinase in dengue virus replication. *Sci. Rep.* **6**, 30490; doi: 10.1038/srep30490 (2016).

## Supplementary Material

Supplementary Information

## References

[b1] Bhatt S. . The global distribution and burden of dengue. Nature 496, 504–507, 10.1038/nature12060 (2013). 23563266 PMC3651993

[b2] Lou Z., Sun Y. & Rao Z. Current progress in antiviral strategies. Trends in Pharmacological Sciences 35, 86–102, doi: http://dx.doi.org/10.1016/j.tips.2013.11.006 (2014). 24439476 10.1016/j.tips.2013.11.006 PMC7112804

[b3] Schwegmann A. & Brombacher F. Host-directed drug targeting of factors hijacked by pathogens. Science signaling 1, re8, 10.1126/scisignal.129re8 (2008). 18648074

[b4] Tan S. L., Ganji G., Paeper B., Proll S. & Katze M. G. Systems biology and the host response to viral infection. Nature biotechnology 25, 1383–1389, 10.1038/nbt1207-1383 (2007). PMC7097743 18066032

[b5] Medigeshi G. R. Mosquito-borne flaviviruses: overview of viral life-cycle and host-virus interactions. Future Virology 6, 1075–1089, 10.2217/fvl.11.85 (2011).

[b6] Foa R. . Dasatinib as first-line treatment for adult patients with Philadelphia chromosome-positive acute lymphoblastic leukemia. Blood 118, 6521–6528, 10.1182/blood-2011-05-351403 (2011). 21931113

[b7] Talpaz M. . Dasatinib in imatinib-resistant Philadelphia chromosome-positive leukemias. The New England journal of medicine 354, 2531–2541, 10.1056/NEJMoa055229 (2006). 16775234

[b8] Yu Y. . Inhibition of Spleen Tyrosine Kinase Potentiates Paclitaxel-Induced Cytotoxicity in Ovarian Cancer Cells by Stabilizing Microtubules. Cancer cell, 10.1016/j.ccell.2015.05.009 (2015). PMC5257279 26096845

[b9] Shimojima M., Ikeda Y. & Kawaoka Y. The Mechanism of Axl-Mediated Ebola Virus Infection. Journal of Infectious Diseases 196, S259–S263, 10.1086/520594 (2007). 17940958

[b10] Lupberger J. . EGFR and EphA2 are host factors for hepatitis C virus entry and possible targets for antiviral therapy. Nature medicine 17, 589–595, 10.1038/nm.2341 (2011). PMC3938446 21516087

[b11] Eierhoff T., Hrincius E. R., Rescher U., Ludwig S. & Ehrhardt C. The epidermal growth factor receptor (EGFR) promotes uptake of influenza A viruses (IAV) into host cells. PLoS pathogens 6, e1001099, 10.1371/journal.ppat.1001099 (2010). 20844577 PMC2936548

[b12] Kumar N., Sharma N. R., Ly H., Parslow T. G. & Liang Y. Receptor tyrosine kinase inhibitors that block replication of influenza a and other viruses. Antimicrobial agents and chemotherapy 55, 5553–5559, 10.1128/AAC.00725-11 (2011). 21930873 PMC3232778

[b13] Kunte D. P. . Down-regulation of the tumor suppressor gene C-terminal Src kinase: An early event during premalignant colonic epithelial hyperproliferation. FEBS Letters 579, 3497–3502, 10.1016/j.febslet.2005.05.030. 15961079

[b14] Chu J. J. & Yang P. L. c-Src protein kinase inhibitors block assembly and maturation of dengue virus. Proc Natl Acad Sci USA 104, 3520–3525 (2007). 17360676 10.1073/pnas.0611681104 PMC1805510

[b15] de Wispelaere M., LaCroix A. J. & Yang P. L. The small molecules AZD0530 and dasatinib inhibit dengue virus RNA replication via Fyn kinase. J Virol 87, 7367–7381, 10.1128/JVI.00632-13 (2013). 23616652 PMC3700292

[b16] Okada M. Regulation of the SRC family kinases by Csk. International journal of biological sciences 8, 1385–1397, 10.7150/ijbs.5141 (2012). 23139636 PMC3492796

[b17] Klinghoffer R. A., Sachsenmaier C., Cooper J. A. & Soriano P. Src family kinases are required for integrin but not PDGFR signal transduction. The EMBO journal 18, 2459–2471, 10.1093/emboj/18.9.2459 (1999). 10228160 PMC1171328

[b18] Nakayama Y. . c-Src but not Fyn promotes proper spindle orientation in early prometaphase. The Journal of biological chemistry 287, 24905–24915, 10.1074/jbc.M112.341578 (2012). 22689581 PMC3408174

[b19] Vang T. . Activation of the COOH-terminal Src kinase (Csk) by cAMP-dependent protein kinase inhibits signaling through the T cell receptor. The Journal of experimental medicine 193, 497–507 (2001). 11181701 10.1084/jem.193.4.497 PMC2195911

[b20] Sondhi D. & Cole P. A. Domain interactions in protein tyrosine kinase Csk. Biochemistry 38, 11147–11155, 10.1021/bi990827+ (1999). 10460171

[b21] Yaqub S. . Activation of C-terminal Src kinase (Csk) by phosphorylation at serine-364 depends on the Csk-Src homology 3 domain. The Biochemical journal 372, 271–278, 10.1042/BJ20030021 (2003). 12600271 PMC1223381

[b22] Kazi J. U. . The tyrosine kinase CSK associates with FLT3 and c-Kit receptors and regulates downstream signaling. Cellular signalling 25, 1852–1860, 10.1016/j.cellsig.2013.05.016 (2013). 23707526

[b23] Yamauchi Y. & Helenius A. Virus entry at a glance. Journal of Cell Science 126, 1289–1295, 10.1242/jcs.119685 (2013). 23641066

[b24] Hahn A. S. . The ephrin receptor tyrosine kinase A2 is a cellular receptor for Kaposi’s sarcoma-associated herpesvirus. Nature medicine 18, 961–966, 10.1038/nm.2805 (2012). PMC3645317 22635007

[b25] Coyne C. B. & Bergelson J. M. Virus-induced Abl and Fyn kinase signals permit coxsackievirus entry through epithelial tight junctions. Cell 124, 119–131 (2006). 16413486 10.1016/j.cell.2005.10.035

[b26] Brindley M. A. . Tyrosine kinase receptor Axl enhances entry of Zaire ebolavirus without direct interactions with the viral glycoprotein. Virology 415, 83–94, 10.1016/j.virol.2011.04.002 (2011). 21529875 PMC3107944

[b27] Garcia M. . Productive replication of Ebola virus is regulated by the c-Abl1 tyrosine kinase. Science translational medicine 4, 123ra124, 10.1126/scitranslmed.3003500 (2012). PMC4794994 22378924

[b28] Cai Y., Liu Y. & Zhang X. Suppression of coronavirus replication by inhibition of the MEK signaling pathway. J Virol 81, 446–456, 10.1128/JVI.01705-06 (2007). 17079328 PMC1797436

[b29] Elbahesh H. . Novel roles of focal adhesion kinase in cytoplasmic entry and replication of influenza A viruses. J Virol 88, 6714–6728, 10.1128/JVI.00530-14 (2014). 24696469 PMC4054363

[b30] Fouquet B. . Focal adhesion kinase is involved in rabies virus infection through its interaction with viral phosphoprotein P. J Virol 89, 1640–1651, 10.1128/JVI.02602-14 (2015). 25410852 PMC4300764

[b31] Liang Y. & Roizman B. State and Role of Src Family Kinases in Replication of Herpes Simplex Virus 1. Journal of Virology 80, 3349–3359 (2006). 16537602 10.1128/JVI.80.7.3349-3359.2006 PMC1440421

[b32] Supekova L. . Identification of human kinases involved in hepatitis C virus replication by small interference RNA library screening. The Journal of biological chemistry 283, 29–36, 10.1074/jbc.M703988200 (2008). 17951261

[b33] Best S. M. . Inhibition of interferon-stimulated JAK-STAT signaling by a tick-borne flavivirus and identification of NS5 as an interferon antagonist. J Virol 79, 12828–12839, 10.1128/JVI.79.20.12828-12839.2005 (2005). 16188985 PMC1235813

[b34] Callaway J. B. . Spleen Tyrosine Kinase (Syk) Mediates IL-1beta Induction by Primary Human Monocytes During Antibody-Enhanced Dengue Virus Infection. The Journal of biological chemistry, 10.1074/jbc.M115.664136 (2015). PMC4498069 26032420

[b35] Ho L. J. . Dengue virus type 2 antagonizes IFN-alpha but not IFN-gamma antiviral effect via down-regulating Tyk2-STAT signaling in the human dendritic cell. Journal of immunology 174, 8163–8172 (2005). 10.4049/jimmunol.174.12.8163 15944325

[b36] Kundu K., Dutta K., Nazmi A. & Basu A. Japanese encephalitis virus infection modulates the expression of suppressors of cytokine signaling (SOCS) in macrophages: implications for the hosts’ innate immune response. Cellular immunology 285, 100–110, 10.1016/j.cellimm.2013.09.005 (2013). 24140964

[b37] Lin R. J., Chang B. L., Yu H. P., Liao C. L. & Lin Y. L. Blocking of interferon-induced Jak-Stat signaling by Japanese encephalitis virus NS5 through a protein tyrosine phosphatase-mediated mechanism. J Virol 80, 5908–5918, 10.1128/JVI.02714-05 (2006). 16731929 PMC1472572

[b38] Meertens L. . The TIM and TAM families of phosphatidylserine receptors mediate dengue virus entry. Cell host & microbe 12, 544–557, 10.1016/j.chom.2012.08.009 (2012). 23084921 PMC3572209

[b39] Levinson N. M., Visperas P. R. & Kuriyan J. The tyrosine kinase Csk dimerizes through Its SH3 domain. PloS one 4, e7683, 10.1371/journal.pone.0007683 (2009). 19888460 PMC2766628

[b40] Hirsch A. J. . The Src family kinase c-Yes is required for maturation of West Nile virus particles. J Virol 79, 11943–11951 (2005). 16140770 10.1128/JVI.79.18.11943-11951.2005 PMC1212629

[b41] Brandvold K. R., Steffey M. E., Fox C. C. & Soellner M. B. Development of a Highly Selective c-Src Kinase Inhibitor. ACS Chemical Biology 7, 1393–1398, 10.1021/cb300172e (2012). 22594480 PMC3423592

[b42] Karaman M. W. . A quantitative analysis of kinase inhibitor selectivity. Nature biotechnology 26, 127–132, 10.1038/nbt1358 (2008). 18183025

[b43] Cao H., Sanguinetti A. R. & Mastick C. C. Oxidative stress activates both Src-kinases and their negative regulator Csk and induces phosphorylation of two targeting proteins for Csk: caveolin-1 and paxillin. Experimental cell research 294, 159–171, 10.1016/j.yexcr.2003.11.010 (2004). 14980511

[b44] Trinh T. B., Xiao Q. & Pei D. Profiling the Substrate Specificity of Protein Kinases by On-Bead Screening of Peptide Libraries. Biochemistry 52, 5645–5655, 10.1021/bi4008947 (2013). 23848432 PMC3773219

[b45] Tourdot B. E. . Immunoreceptor Tyrosine-Based Inhibitory Motif (ITIM)-Mediated Inhibitory Signaling Is Regulated by Sequential Phosphorylation Mediated by Distinct Nonreceptor Tyrosine Kinases: A Case Study Involving PECAM-1. Biochemistry 52, 2597–2608, 10.1021/bi301461t (2013). 23418871 PMC3666314

[b46] Agrawal T., Schu P. & Medigeshi G. R. Adaptor protein complexes-1 and 3 are involved at distinct stages of flavivirus life-cycle. Scientific reports 3, 1813, 10.1038/srep01813 (2013). 23657274 PMC3648799

[b47] Kakumani P. K. . Role of RNA Interference (RNAi) in Dengue Virus Replication and Identification of NS4B as an RNAi Suppressor. Journal of Virology 87, 8870–8883, 10.1128/jvi.02774-12 (2013). 23741001 PMC3754049

[b48] Haridas V. . Bispidine-Amino Acid Conjugates Act as a Novel Scaffold for the Design of Antivirals That Block Japanese Encephalitis Virus Replication. PLoS Negl Trop Dis 7, e2005, 10.1371/journal.pntd.0002005 (2013). 23350007 PMC3547849

