# Supplementary material for: Identification and characterization of the role of c-terminal Src kinase in dengue virus replication
Source: Sci Rep. 2016 Jul 26;6:30490. doi: 10.1038/srep30490 (PMC4960526; doi:10.1038/srep30490)
Supplement: Supplementary Information [file srep30490-s1.pdf]

# **Identification and characterization of the role of c-terminal Src kinase in dengue virus replication**

Rinki Kumar, Tanvi Agrawal, Naseem Ahmed Khan, Yuji Nakayama and Guruprasad R. Medigeshi

## SUPPLEMENTARY FIGURE LEGENDS

**Fig. S1. Identification of TKs that inhibit or enhance DENV infection using siRNA screening.** (A and B) Huh-7 cells were transfected with 10 nM each of smartpool siRNAs targeting the 88 human TKs and 48 h post-transfection, cells were infected with 1 MOI of DENV-2. Viral titers in the infected culture supernatants were measured at 24 h pi by plaque assay. Red line indicates 50% titer values relative to non-targeting control (C) siRNA and green line indicates a two-fold increase threshold in viral titers relative to control siRNA. Data are from two experiments performed with two or three replicates and indicate mean with SEM. Arrows indicate the five inhibitory tyrosine kinases shown in figure 1.

**Fig. S2. Csk knock-down inhibits DENV replication in BHK-21 cells.** (A) BHK-21 cells were transfected with 10 nM of siRNAs targeting *CSK* or non-targeting control (NTC) and 48 h post-transfection, cells were infected with 1 MOI of DENV-2. Viral titers in the infected culture supernatants were measured at 24 h pi by plaque assay. The data are representative of two experiments performed with three replicates and indicate mean with SEM. P value was calculated by unpaired t test. ****$p<0.0001$.

**Fig. S3. Csk knock-down does not block DENV internalization.** Huh-7 cells were transfected with 10 nM of either of the two siRNAs targeting *CSK* and 48 h post-transfection, cells were infected with 5 MOI of DENV-2. Total RNA was isolated 1 h post-infection and the amount of DENV genome inside the cells were measured by real-time PCR. Fold change was calculated by normalizing DENV Ct values with β-

actin transcript levels. The data are representative of two experiments performed with three replicates and indicate mean with SEM.

**Fig. S4. The kinase domain and SH3 domain of Csk is important for DENV infection.** (A) Huh-7 cells were transfected with the plasmids for overexpression of the indicated proteins tagged with a FLAG epitope for detection. 24 h post-transfection, cells were infected with 5 MOI of DENV-2. Cells were fixed at 24 h pi and stained using primary antibodies against DENV-Envelope and FLAG epitope followed by secondary antibodies conjugated with Alexa-488 (green) and Alexa-568 (Red) respectively. Nuclei were stained with DAPI. UT- Untransfected. Scale – 100 μm.

Figure S1

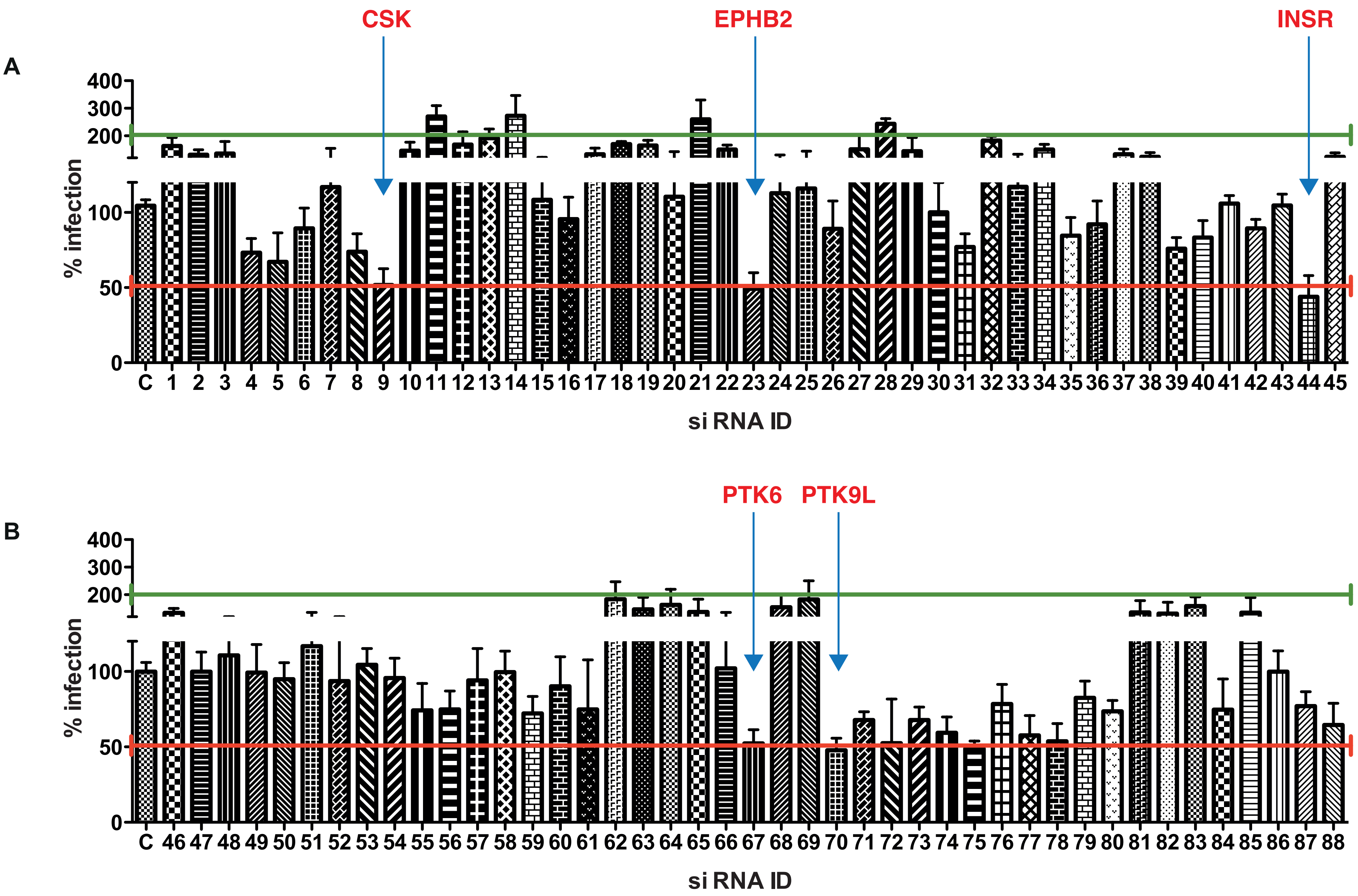

A
CSK
EPHB2
INSR
400
300
200
100
50
0
% infection
C 1 2 3 4 5 6 7 8 9 10 11 12 13 14 15 16 17 18 19 20 21 22 23 24 25 26 27 28 29 30 31 32 33 34 35 36 37 38 39 40 41 42 43 44 45
si RNA ID
B
PTK6
PTK9L
400
300
200
100
50
0
% infection
C 46 47 48 49 50 51 52 53 54 55 56 57 58 59 60 61 62 63 64 65 66 67 68 69 70 71 72 73 74 75 76 77 78 79 80 81 82 83 84 85 86 87 88
si RNA ID

**Figure S2**

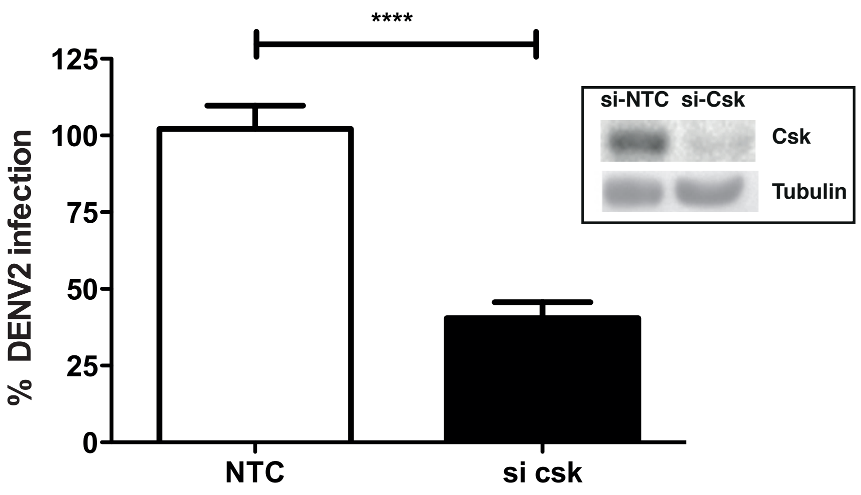
****
125
100
75
50
25
0
% DENV2 infection
NTC
si csk
si-NTC
si-Csk
Csk
Tubulin

**Figure S3**

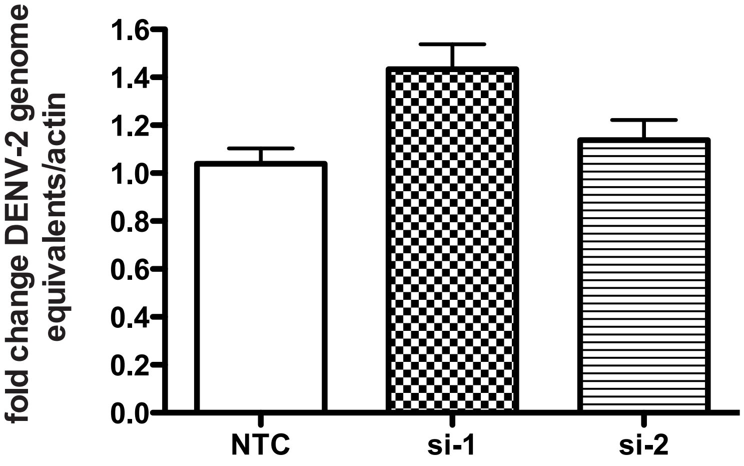
fold change DENV-2 genome equivalents/actin
1.6
1.4
1.2
1.0
0.8
0.6
0.4
0.2
0.0
NTC
si-1
si-2

## Figure S4

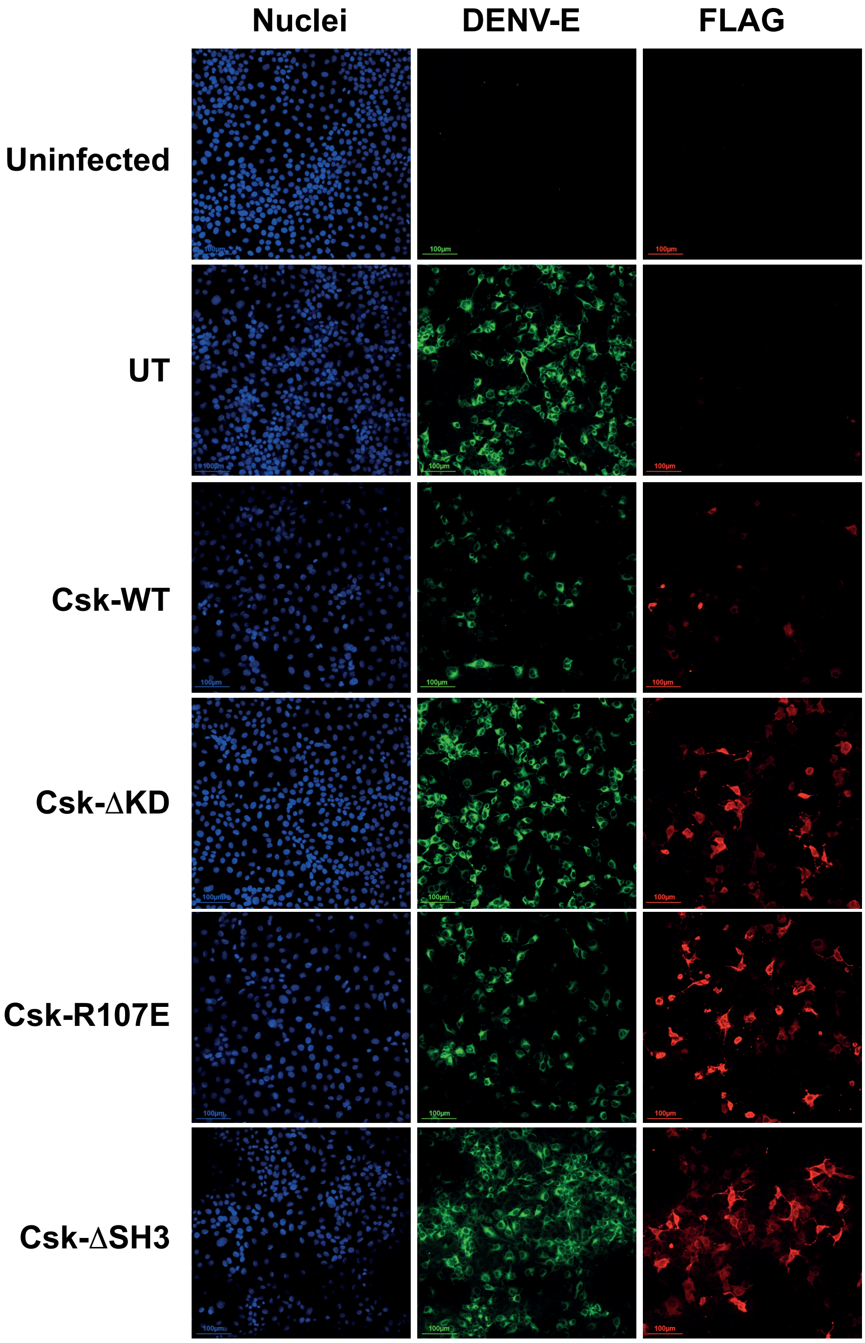

Nuclei
DENV-E
FLAG
Uninfected
UT
Csk-WT
Csk-ΔKD
Csk-R107E
Csk-ΔSH3
